# Multilayer microhydraulic actuators with speed and force configurations

**DOI:** 10.1038/s41378-021-00240-7

**Published:** 2021-03-11

**Authors:** Jakub Kedzierski, Hero Chea

**Affiliations:** Massachusetts Institute of Technology Lincoln Laboratory, Lexington, MA 02420 USA

**Keywords:** Electrical and electronic engineering, Electronic properties and materials

## Abstract

Electrostatic motors have traditionally required high voltage and provided low torque, leaving them with a vanishingly small portion of the motor application space. The lack of robust electrostatic motors is of particular concern in microsystems because inductive motors do not scale well to small dimensions. Often, microsystem designers have to choose from a host of imperfect actuation solutions, leading to high voltage requirements or low efficiency and thus straining the power budget of the entire system. In this work, we describe a scalable three-dimensional actuator technology that is based on the stacking of thin microhydraulic layers. This technology offers an actuation solution at 50 volts, with high force, high efficiency, fine stepping precision, layering, low abrasion, and resistance to pull-in instability. Actuator layers can also be stacked in different configurations trading off speed for force, and the actuator improves quadratically in power density when its internal dimensions are scaled-down.

## Introduction

The invention of electrostatic motors, pioneered by Benjamin Franklin and Andrew Gordon in 1740, significantly predates Michael Faraday’s demonstration of the first inductive motor in 1821, yet electrostatic motors have never gained a significant technological foothold. Historically, electrostatic motors have required high voltage and had low output power. In the last few decades, microelectromechanical (MEMS) motors^1^ have improved the outlook for capacitively driven rotational actuation. At the microscale, a higher driving frequency can increase the power density, and smaller electrode gaps can reduce the driving voltage. Some high-frequency piezoelectrically driven ultrasonic motors^2,3^ have gained commercial acceptance; however, most MEMS motors still suffer from an unacceptably low torque and the inability to scale in three dimensions due to their inherently thin nature^4^. To address these challenges a desirable electrostatic motor technology should offer a low operating voltage, high torque, high efficiency, and the ability to scale up in thickness. It is known how to achieve these characteristics individually. A low-voltage operation can be obtained by using a thin dielectric^5,6^. A high torque can be obtained by having a large capacitance change in a small displacement, either by using planar capacitive coupling^5,7^ or by using a small stepping distance^5,8,9^. Extendibility in thickness, without increasing voltage, can be obtained with a layered structure design^10,11^. Finally, high efficiency can be obtained by using a dielectric with low loss^5,8,12^. All these characteristics have been individually demonstrated, but to our knowledge, they have never been combined into a single actuator technology.

In this work, we demonstrate such a technology by extending microhydraulic electrowetting actuators^5,13^, which already demonstrate low voltage, high-torque density, and high efficiency, into the third dimension by layering multiple actuating films into a single unit. Microhydraulic technology works by electrically distorting the equilibrium surface tension state of attached liquid droplets with electrowetting^14–17^. In previous work, droplet arrays on a thin film of polyimide^5,13^ were actuated by a dielectric-covered electrode array on top of a thick base. To extend this technology in three dimensions, we integrated the electrode and droplet arrays into one thin film, with the droplet array on one side and the electrode array on the other. The resulting layers can be stacked and individually powered throughout the stack with a liquid interconnect network^18,19^. In addition to high torque, low voltage, efficiency, and stacking, our actuators have four other desirable qualities. First, all solid moving components are separated by a fluidic layer and never come into direct contact, thus avoiding stiction and abrasion issues that are common in MEMs motors^20^. Second, the dielectric is rigid; thus, the electric field during actuation remains largely constant for charged regions, avoiding pull-in instability issues that cause breakdown in compliant dielectric actuators^21^. Third, as will be shown in this work, different configurations can be used to internally gear the actuator, trading off speed for torque, as the applications demand. Fourth, the power and force densities scale quadratically as the internal size scale of the actuator is reduced, giving it a Moore’s Law-like scaling advantage^5^. Even with the modest droplet pitch of 40 µm, we demonstrate a power density similar to inductive motors at much higher torque. Scaling to a 15 µm pitch gives roughly another order of magnitude in torque and power. Due to this unique combination of characteristics, multilayer microhydraulic actuators can foster future advances in responsive robotic joints, microrobotics, and robotic surgery.

## Results

### Structure

**Fig. 1:**
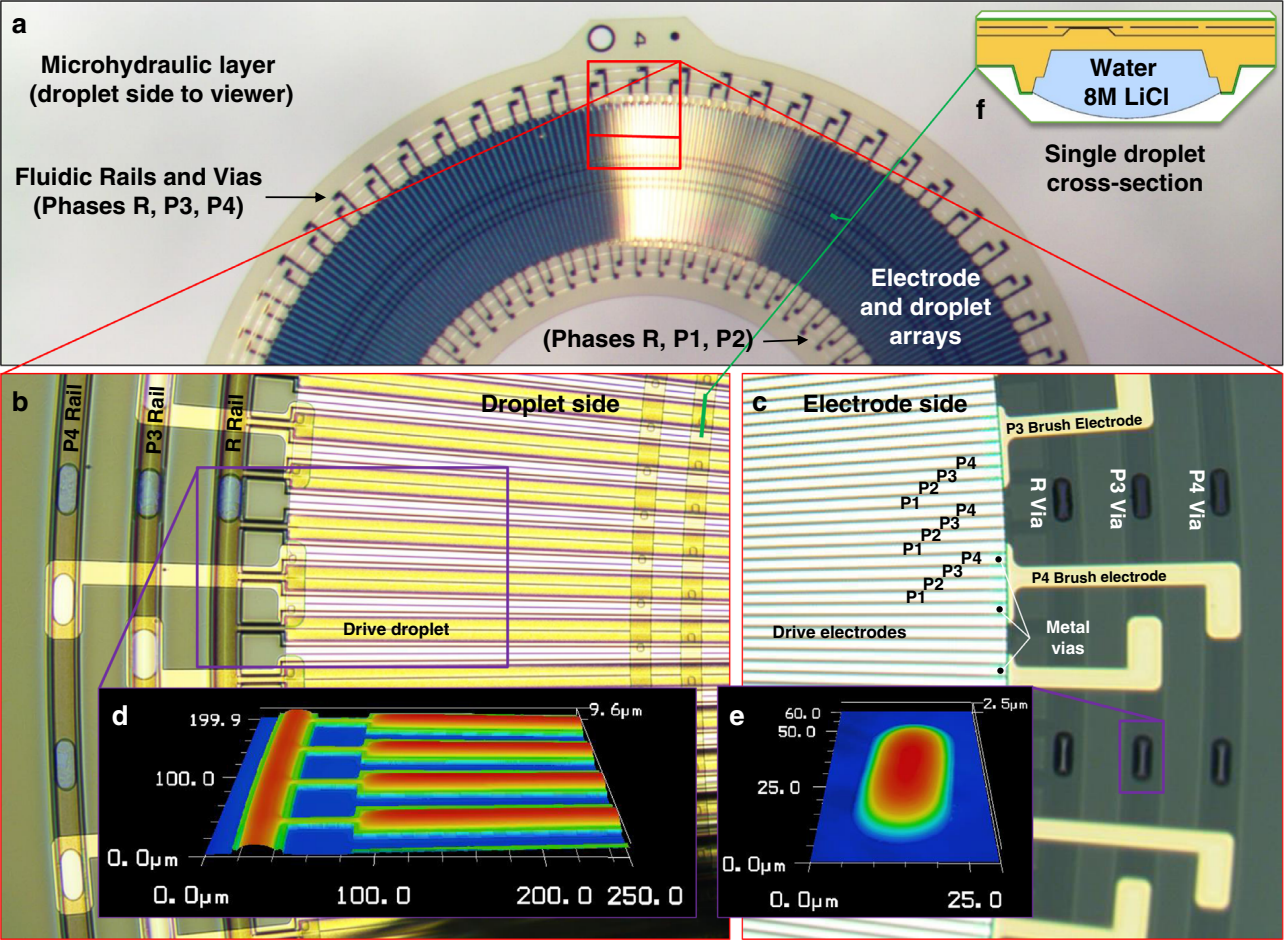
A detailed view of a single microhydraulic rotational layer. **a** A low magnification picture taken from the droplet side, showing the outside rails, inside rails, the droplet array, and the electrode array. **b** A magnified view of the droplet side with the drive droplets and outside rails. Features on the electrode side are also visible since polyimide and water are transparent. **c** Electrode side with the four-phase Al drive electrodes, and the Pt brush electrodes. **d** Confocal microscope height map of the droplet side, showing the surface curvature of the drive and rail droplets as well as the edge of the droplet wall. **e** A height map of the fluidic via from the electrode side. Liquid profiles on both sides have a radius curvature of ~18 µm, which corresponds to a Laplace pressure of 3.8 kPa or 31 cm of 8 M LiCl with the air/water surface tension. Curvature is similar to that caused by the 24 cm of 8 M LiCl used in the fabrication of the actuator with the oil/water surface tension. A cross-section diagram of a cycle section of the actuator is shown in **f**. Rotational microhydraulic layers were 10-µm thick and had a 6-mm outer diameter.

The actuators consisted of stacked 10-µm-thick polyimide layers separated by fluid. The layers were functionalized on both sides, one side had electrowetting^5,13^ electrodes covered by a dielectric, and the other side had hydrophilic regions wetted with long linear water droplets. The actuators worked by having layers move past each other when electrodes on one layer attracted the droplets on the layer above it^5^. Figure 1 shows a detailed view of a single rotational layer, viewed from the droplet and electrode sides. The droplet side, shown in Fig. 1a, b, d, consisted of hydrophilic and hydrophobic surfaces, with hydrophilic areas wetted by water containing 8 M LiCl, forming linear semicylindrical droplets. Uniform Laplace pressure gave connected droplets a specific curvature, as shown in Fig. 1d, e. The two types of droplets, radial and circumferential, served different functions. The radial “drive” droplets were responsible for interlayer motion when distorted by electrowetting forces, and formed the 330 (*N*_drop_) unit circular droplet array. The circumferential “rail” droplets formed the three inner and three outer rails, which carried the electrical signals for the stepping phases P1-P4, and reference potential *R*, at which the drive droplets were held. These rails also self-aligned layers in X, Y, and Z during assembly. The electrode side, shown in Fig. 1c, e, consisted of Al and Pt metal layers. The Al layer formed the drive electrodes and was covered by an electrowetting dielectric of 1.1 µm of polyimide and 14 nm of fluoropolymer. The Pt brush electrodes connected the drive electrodes to the fluidic rails of the right phase. Pt was required so that no corrosion or oxidation occurred at the metal–water interface. The fluidic vias, shown wetted in Fig. 1e, connected the rails between layers, forming the electrical conduction path between the rails in each layer and the base. The order of the drive electrodes alternated from P1 to P4 in a counterclockwise manner for the regular electrode-order layers and in a clockwise manner for the inverted electrode-order layers. Each unit of four electrodes and one drive droplet formed a repeating cycle segment of the actuator, giving 330 cycles for a complete rotation, and 1340 driving electrodes per rotational layer. The length of a cycle segment was one droplet pitch (*D*_pitch_) of 1.09° or ~40 µm in the middle of the array. Finally, there were two special layers, namely, the base and the top. The base had no driving electrodes or driving droplets and consisted only of the six rails and external electrical connections (see the “Liquid interconnect” section for more details). The top layer had no driving electrodes with only logo text in the metal layer.

**Fig. 2:**
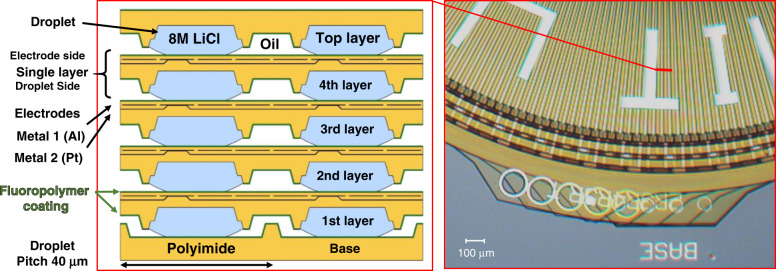
A cross-sectional view (left) and a top-down micrograph (right), of a stacked five-layer microhydraulic rotational actuator. The first layer sits in a base and does not move, subsequent layers move by having the drive droplets glide on the fluoropolymer coating of the drive electrodes in the layers below. The layer tag numbers for layers 1–5 are 2R, 3R, 4R, 5R, and T, respectively, from the bottom to the top. Layers 2–5R have inverted electrode order (indicated by the R); with the final layer T displaying the MIT Logo text. Dodecane oil surrounds the entire actuator and forms the insulating ambient fluid between layers.

The layers were stacked in an oil bath, typically dodecane. The stacked-layer structure for a five-layer rotational actuator is shown in Fig. 2, with a base layer at the bottom and a top layer on the top. The first layer was stacked on the base, droplet side to droplet side, with the water rails bridged for each phase. Neither the base nor the first layer moved. Subsequent moving layers were stacked droplet side to electrode side, with the fluidic rails aligning to the fluidic vias of the layer below, and the driving droplets aligning to the driving electrodes. Surface tension forces between fluidic vias and rails self-aligned the layers into translational and vertical alignment^22^, while rotational alignment was achieved when P2 and P3 were held high, typically at 30 V. Before self-alignment was possible, the layers had to be manually aligned to within half rail pitch (40 µm) translationally, and within half cycle angle (0.6°) rotationally. After self-alignment, the translational misalignment was <1 µm, and rotational misalignment was <0.03°. The rotational layers were 6 mm in diameter, and the layer-to-layer pitch was 15 µm. This gave the rotational actuator, consisting of five layers and a base, a thickness of only 82 µm, and a volume of only 2.3 µL. Linear actuators were built in a similar way using rectangular 2.7 by 3.0 mm layers.

### Operation

**Fig. 3:**
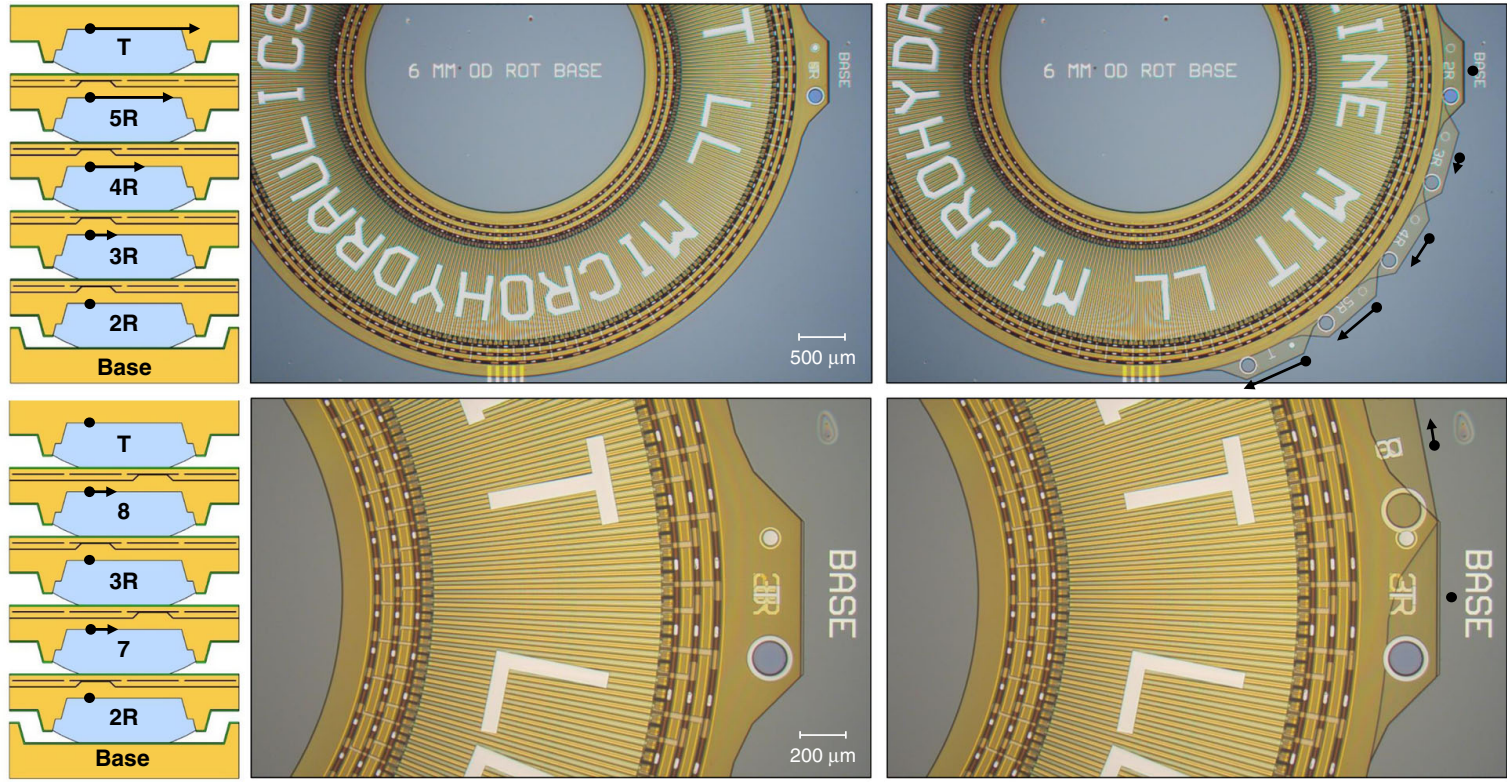
Images of five-layer stack actuation in the speed (top) and force (bottom) configurations, left column also shows the cycle cross-section profile for the corresponding configuration. Absolute velocity vectors are indicated for each layer with an arrow next to the layer tab. For the speed configuration (with layer order 2R, 3R, 4R, 5R, and T), each subsequent layer moves with a fixed velocity (F_cyc_D_pitch_) relative to the layer below it. As a result, the fifth layer (T) moves four times as fast as the second layer (3R). In the force configuration (with layer order 2R, 7, 3R, 8, T), each layer moves in the opposite direction relative to the one below it. Relative to the base, the odd layers (2R, 3R, T) remain stationary, while the even layers (7, 8) move at a uniform velocity (F_cyc_D_pitch_). Movies of these actuations are available in the Supplementary Materials.

Microhydraulic actuator operation for rotational and linear actuators is described in detail in our previous work^5^. To summarize, during actuation, square-wave voltage signals up to 50 V were sent to each phase, P1–4, with a quarter-cycle time offset. The signals were 50% duty cycle, and consequently, two out of the four phases were on at any particular time. The reference phase R was biased with a constant zero or a negative voltage down to −20 V. As each new phase was energized, the drive droplet on the layer above was attracted to it and moved to a new position dragging the layer above along. Phase progression from P1–4 resulted in actuator motion in one direction, and phase progression of P4–1 resulted in motion in the opposite direction. Since all layers shared the same rail network, the electrical phase sequence was the same for all layers at any one time. However, the physical electrode order, regular or inverted, depended on how each layer’s drive electrodes connected to the rails and did not have to be the same for all layers. Different physical configurations were used; the simplest configuration, when all layers share the same electrode order, is shown at the top of Fig. 3 for a five-layer actuator. In this configuration, each layer moved in the same direction and with the same velocity relative to the layer below it. Relative to the first stationary layer, the second layer moved at a velocity of *F*_cyc_*D*_pitch_, the next layer moved at 2*F*_cyc_*D*_pitch_, and so on. Because the speed increased with the number of layers, as (*N*_layer_ − 1)*F*_cyc_*D*_pitch_, we refer to this configuration as the speed configuration. The blocked force, or torque, was independent of layer number, as only the last layer pushed with a force equal to that of a two-layer actuator. That force was ~*N*_drop_*W*_drop_*λ*^5^, where *N*_drop_ is the number of drive droplets (330), *W*_drop_ is the individual drive droplet width of 0.9 mm, and *λ* is the surface tension of the oil/water interface, measured at 40 mN/m. A different five-layer configuration is shown at the bottom of Fig. 3. In this configuration, the physical electrode order alternated between regular and inverted as layers were added to the stack. As a result, each layer moved in the opposite direction relative to the one below it. Relative to the base, the odd (inverted electrode order) layers stayed stationary, while the even (regular electrode order) layers moved at a velocity of *F*_cyc_*D*_pitch_. Adding more layers in this configuration does not change the velocity; however, each additional layer adds to the available force since all the layers push in unison. With only two layers, the force is *N*_drop_*W*_drop_*λ* but an added third layer also pushes the second layer from the top, giving a three-layer actuator a force of 2*N*_drop_*W*_drop_*λ*. Since the force scales as (*N*_layer_ − 1)*N*_drop_*W*_drop_*λ*, we refer to this configuration as the force or torque configuration. Importantly, the power available per layer is the same regardless of the configuration since the total layer capacitance, voltage, and frequency remain constant. Actuation movies for both configurations are available in the Supplementary Material.

**Fig. 4:**
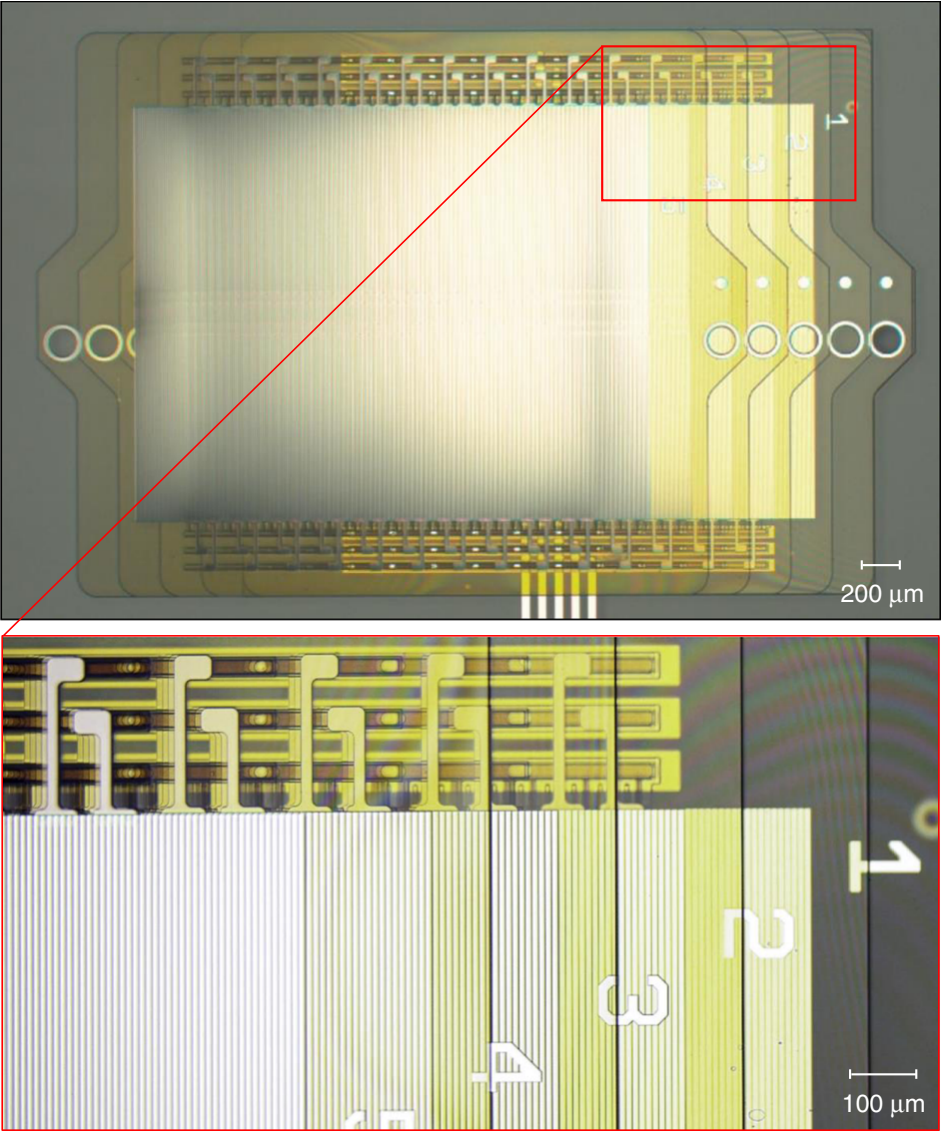
An image of a five-layer linear microhydraulic actuator in the speed configuration. In this example, the actuator translates 19 steps per layer from initial position, one step short of the brush electrode pitch of five cycles. A movie of this actuation is available in the Supplementary Materials.

The actuation of a five-layer linear actuator in a speed configuration is shown in Fig. 4. Linear devices worked in a similar manner to the rotational actuators and had a *W*_drop_ of 2 mm, *N*_drop_ of 50, and *D*_pitch_ of 40 µm. The main operational difference was that unlike rotational actuators, linear ones do not have an infinite stroke, and edge conditions at the end of the arrays are important. Since the droplet array should remain on top of the layer below at all times, the effective stroke of these actuators was approximately ten cycles in either direction. A movie of this actuator is available in the Supplementary Material.

### Metrics

Using the moment arm length, *R*_mean_ of 2.2 mm, the blocked torque of a five-layer actuator in the force configuration can be calculated as (*N*_layer_ − 1)*R*_mean_*N*_drop_*W*_drop_*λ* = 0.11 mNm, giving a torque density of 78 Nm/kg. The angular velocity depended on the maximum cycle frequency, *F*_max_, which in turn depended on the operating voltage. With a P1–4 voltage of 50 V and *R* voltage of −20 V, *F*_max_ was 2000 Hz. At higher frequencies, the actuator occasionally skipped steps. Calculating the angular velocity as *F*_max_*D*_pitch_ gives 38 rad/s for the force configuration. For the five-layer actuator in the speed configuration, the torque was *R*_mean_*N*_drop_*W*_drop_*λ* = 0.026 mNm, and the velocity was (*N*_layer_ − 1) *F*_max_*D*_pitch_ = 152 rad/s. At maximum frequency, the actuator accelerated to full velocity in a single step, or in less than 0.125 ms, which corresponds to an angular acceleration over 0.3 Mrad/s^2^ for a two-layer actuator and 1.2 Mrad/s^2^ for a five-layer actuator. A movie of an actuation at *F*_max_ for a two-layer rotational actuator is available in the Supplementary Material. The maximum output power for either configuration was ~¼*F*_max_*D*_pitch_*N*_drop_*W*_drop_*λ*(*N*_layer_ − 1)^5^, and when normalized by mass, it gave a 0.74 kW/kg output power density, similar to inductive motors.

## Discussion

### Arbitrary configurations

**Fig. 5:**
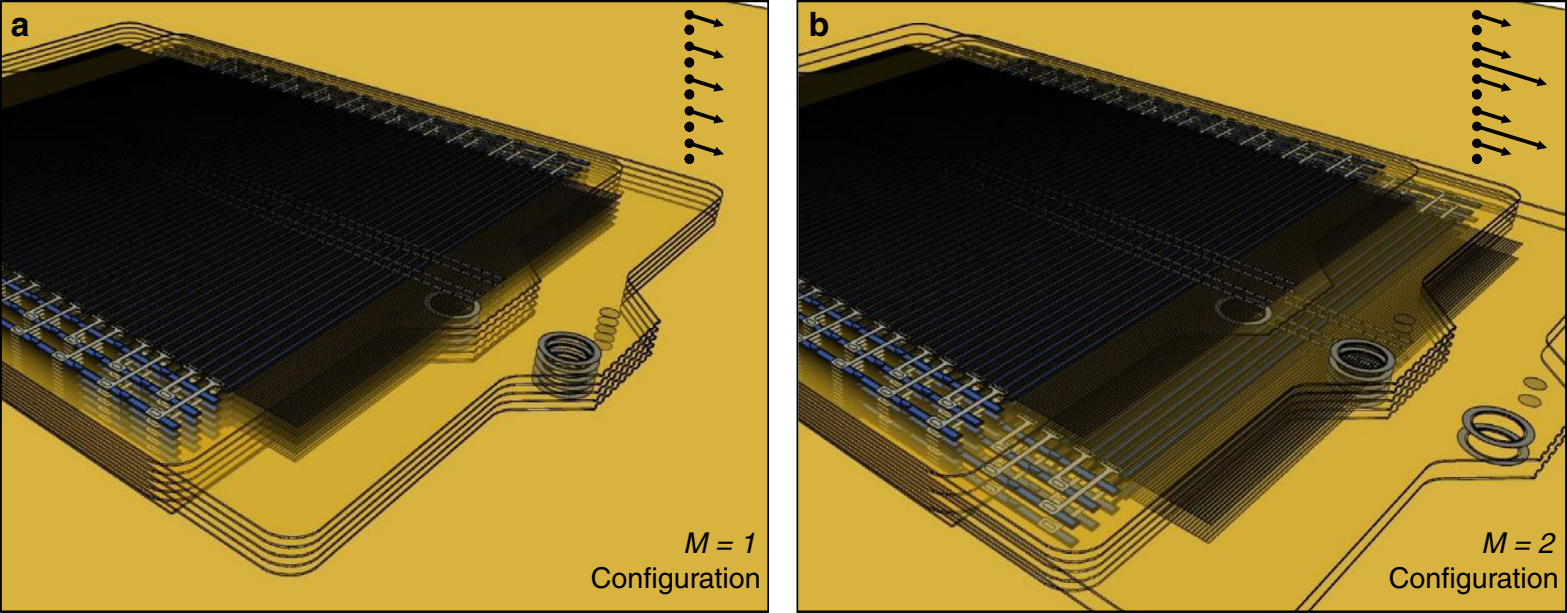
A diagram of two different stacking configurations. The high force *M* = 1 configuration is shown in (**a**), and the *M* = 2 configuration is shown in (**b**). Absolute velocity vectors for each layer are shown with stacked arrows. For any value of *M*, the stacking order will alternate, with *M* forward going layers stacked on top of *M* backward going layers. In general, any *M* configuration will have *M* maximum velocity and 1/*M* force density of the *M* = 1 or force configuration.

The speed and force configurations described in the previous section are only a subset of possible layer arrangements. In general, a multilayer stack can consist of M regular, and M inverted electrode-order layers, alternating up through the actuator. For *M* = 1, this results in the force configuration, shown for a linear actuator in Fig. 5a, but for *M* = 2, shown in Fig. 5b, the actuator has twice the speed at half the force of the *M* = 1 configuration. Generally, for any *M* configuration, the actuator will have 1/*M* force and *M* speed of the force (*M* = 1) configuration. Such design flexibility allows for a tailored speed vs. power profile without the use of gears. This is particularly important for microactuation, where gearing can be difficult, inefficient, and consume a significant amount of size, weight, and power.

### Torque and speed space

**Fig. 6:**
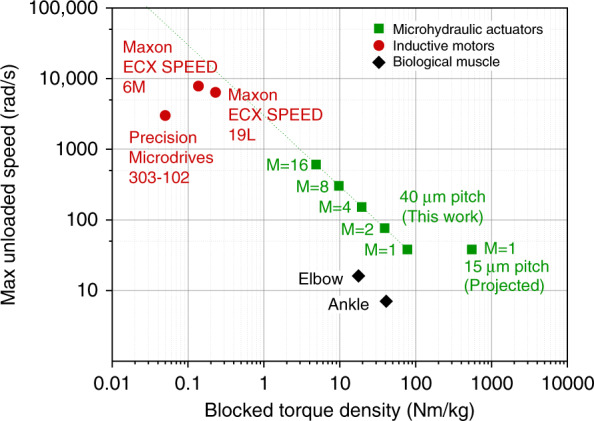
A plot of the maximum unloaded rotational velocity and blocked torque density for various rotational actuators. Inductive motors tend to have a high speed at a low torque density, while microhydraulic motors and biological joints tend to have a low velocity and a high-torque density. Different *M* configurations can exchange speed for torque. The 40 µm droplet pitch devices are shown in this work, while the 15 µm droplet pitch devices are projected from scaling trends. Metrics for the Microdrive 303–102 motor were measured in our laboratory, Maxon motor metrics were taken from online datasheets. Elbow and ankle measurements were obtained from the first author using a load cell and a gyroscope and are typical of biological muscle performance. Normalization masses for the muscle torque calculations were the arm mass below the shoulder, and the leg mass below the knee.

Although the microhydraulic rotational actuators shown in this paper have a similar mechanical power density to inductive motors, when this power density is broken down into torque and speed, a significant difference becomes apparent. Figure 6 shows the maximum unloaded speed vs. the blocked torque density for our actuators, selected high-performance inductive motors, and biological joints. Inductive motors have a high speed and a low torque density, while microhydraulic actuators and biological joints have a much higher torque density and a lower speed. For microhydraulic actuators, the gap can be bridged by using different configurations, but a configuration with *M* over 100 is required to reach the speeds of high-performance inductive motors. The fact that microhydraulic actuators reside in the same region of the torque/speed diagram as biological joints, but are significantly better in both metrics, suggests robotic and micro-robotic applications. Many robotic systems use inductive servos that have a high gear ratio to provide higher torque. Microhydraulic actuators would not need gears and as such could be made more compact and responsive. For example, fabricating a robotic human-like hand is very difficult for current actuator technology, partly due to the lack of space in the fingers for an inductive motor and gears. High-torque microhydraulic actuators could be the solution. Additionally, it is important to point out that microhydraulic actuators can be greatly improved by scaling dimensions, while inductive motors get worse when scaled down. For a droplet pitch of 15 µm, the projected power density is an order of magnitude greater than high-performance inductive motors, at a very high-torque density, as shown in Fig. 6.

### Liquid interconnect

**Fig. 7:**
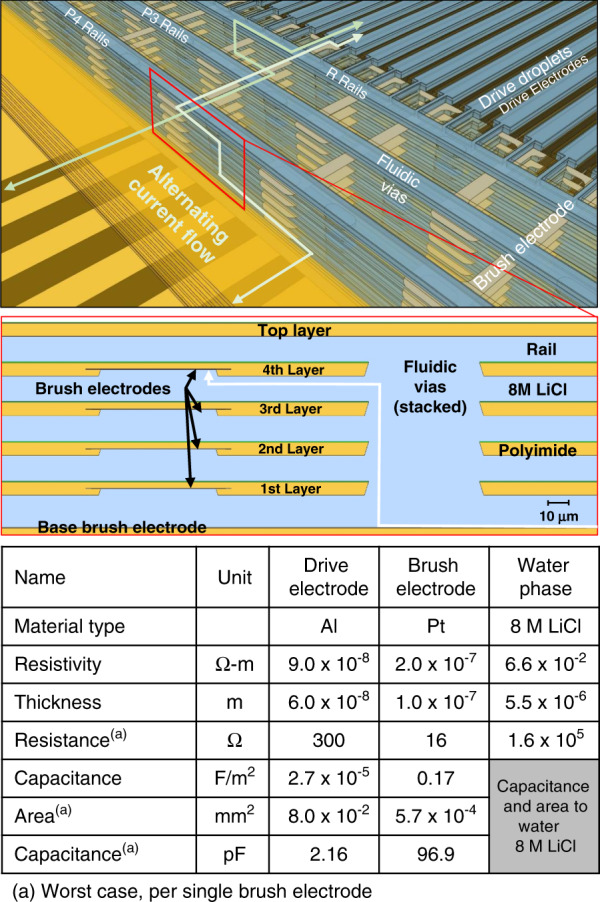
A diagram showing the electrical power distribution network for a linear multilayer microhydraulic actuator. Alternating current flows from the base up through the fluidic rails and vias to the brushes, then to the drive electrodes. It then couples to the drive droplets in the layer above and returns through the fluidic rails and vias back to the base. Driving (Al) electrodes are shown in black, brush (Pt) electrodes are shown in gray, and liquid interconnect components (water 8 M LiCl, rails, droplets, and vias) are shown in blue. The inset shows the cross-section through the fluidic via and the brush electrode double layer. The table shows the measured network parameters with the resistance and capacitance normalized to a subunit of the actuator containing a single brush electrode.

Electrical power had to be connected to all microhydraulic layers, even though they moved relative to each other. This could be done capacitively^13^ for some configurations, but in this work, a liquid interconnect network was used^18,19^. The electrical power delivery path in the actuator is outlined in Fig. 7. Signals enter from the connector to the Al interconnect at the base, connect with metal via to the Pt base brush electrodes, capacitively couple to the water 8 M LiCl rails through an electrical double layer, ionically conduct through the fluidic rails, and vias to the Pt brush electrodes in each layer, transfer through a metal via to the Al drive electrodes, couple capacitively to the drive droplets to form the electrowetting capacitor, then return through the 8 M LiCl reference rail into the *R* Pt brush electrodes in the base, and return to the connector. This complex path requires that no double layer in the system exceeds the electrolysis voltage of 1.2 V; thus, the areas and capacitances of the double layers used for the Pt–liquid connection are critical. The table in Fig. 7 shows the measured electrical parameters of the interconnect network. The double-layer capacitance per area was quite high at 0.17 F/m^2^. The total double-layer capacitance, calculated using the water/Pt contact area of all the brush electrodes, was ~50 times higher than the electrowetting capacitance. This capacitive ratio kept 98% of the applied voltage across the electrowetting dielectric and prevented electrolysis at the Pt brush electrodes. Electrolysis did occur if two phases somehow shorted to each other. Resistance was another potential issue. Even though the water with 8 M LiCl is a relatively low resistance ionic conductor, it is still over five orders of magnitude more resistive than a typical interconnect metal, making it the predominant resistive component in the network. Ionic resistivity, however, did not limit actuator performance, as the resistive–capacitive product was 0.34 µs and thus much shorter than the minimum stepping time of 125 µs.

### Challenges

Microhydraulic technology has unique challenges due to its fluidic nature and reliance on unsupported thin films. In this study, layers had to be low stress to not curl or warp. Careful optimization of the polyimide anneals was required to achieve a radius of curvature for individual layers greater than 10 cm. The fluidic challenges included evaporation and contamination. Evaporation of the water phase was controlled by adding 8 M LiCl. LiCl, being highly deliquescent, prevents water from evaporating at an ambient of 20 °C and 50% relative humidity. In fact, we have a microliter size droplet of water with 8 M LiCl in our lab, exposed to ambient conditions, that has remained largely unchanged for over three years. The dodecane used for the oil phase evaporated slowly from the actuator bath and had to be replenished every few days. In the future, a packaging solution will be required to keep the actuator liquids contained. Impurities in the fluids are also a concern. Molecular impurities, even at low concentrations, can disturb the surface tension and affect actuator performance. To avoid molecular impurities, clean components and fluids were used. Macroparticles of various sizes were an even larger challenge. The liquid-air interfaces of the droplets exposed during wetting, pressurization, release, and assembly tend to grab particles out of the atmosphere and were impossible to clean with techniques we tried. During operation, particles larger than 10 µm often prevented the actuator from working completely by bridging droplets and shorting phases, and smaller particles (1–10 µm) scratched the surface of the electrodes and eventually led to actuator failure. To suppress particle issues, the entire process flow occurred in a clean room. Nevertheless, particles were still the leading cause of yield failure.

## Materials and methods

Actuator fabrication consisted of over 90 steps listed in detail in the Supplementary Material. Below is a summary of the fabrication flow, divided into two consecutive parts: traditional silicon wafer-based processing and custom microhydraulic processing.

### Part 1: Wafer-based processing

**Fig. 8:**
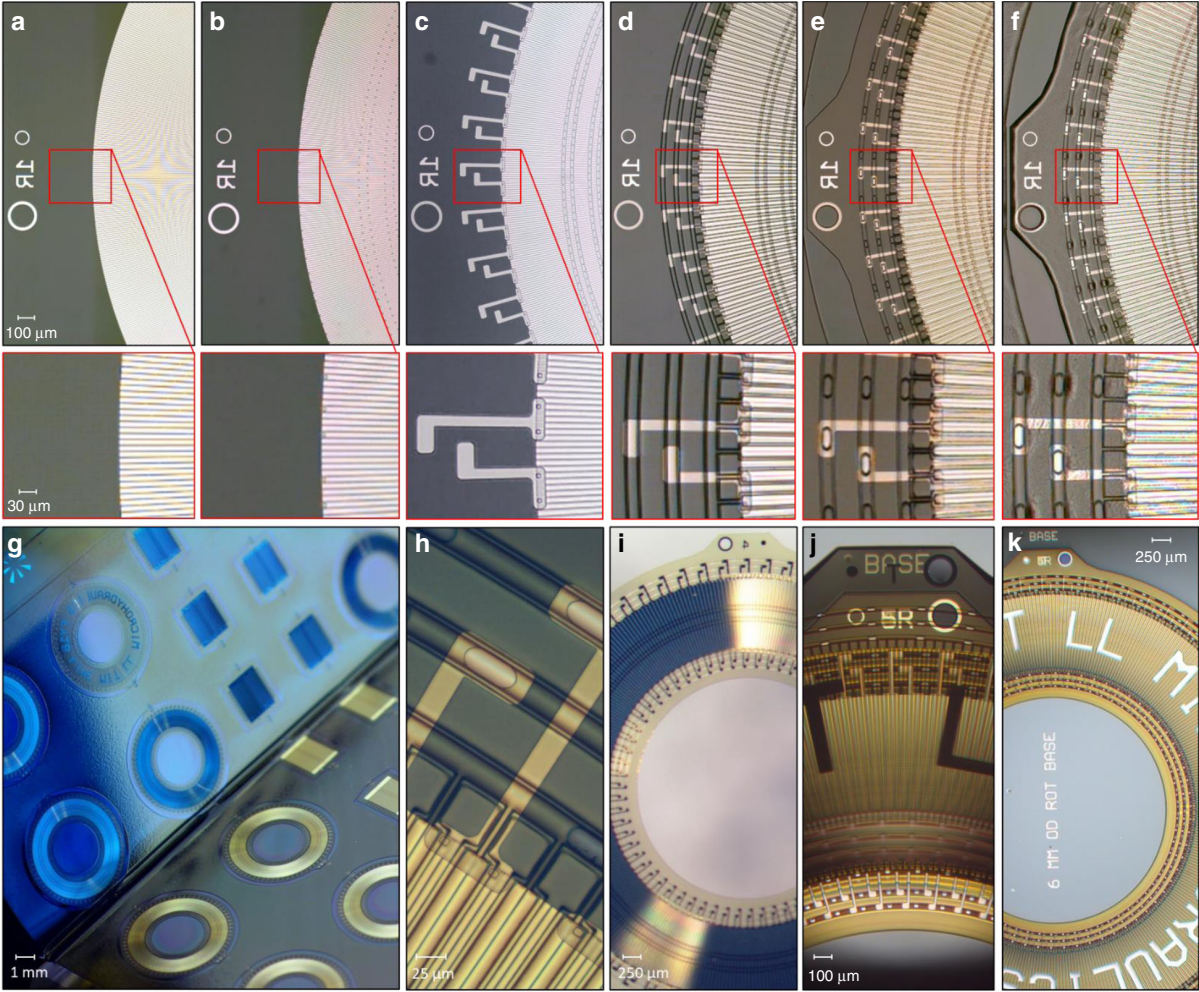
Images of the multilayer microhydraulic actuator at various stages of fabrication. Top row shows the wafer-based processing for all major lithography steps: Al metal (**a**), metal via (**b**), Pt metal (**c**), droplet wall (**d**), outside and die etch (**e**), and hydrophilic patterning (**f**). Bottom row shows the major custom microhydraulics steps: the peel and laydown for the electrowetting fluoropolymer coating and the addition of release wax (**g**), droplet wetting and pressurization (**h**), layer release (**i**), actuator assembly (**j**), and the finished actuator in a testing dodecane bath (**k**). Peel and wetting videos are available in the Supplementary Materials.

The first part of fabrication, outlined in Fig. 8a–f, took place in a 200-mm-silicon wafer fabrication facility (MIT Lincoln Laboratory’s Microelectronics Laboratory). In general, the actuators were fabricated from the electrode side to the droplet side, except for the electrowetting fluoropolymer, which was custom processed last. Fabrication started with cleaned silicon wafers, which were coated with polyimide (Low stress PI2611, DuPont), and cured to give a 1.1-µm-thick film. The drive electrode metal (60 nm Al) was then deposited, patterned, and etched to form the drive electrodes, as shown in Fig. 8a. The interlayer dielectric of 0.9 µm polyimide was spun on and cured. The via layer was then patterned and etched into the polyimide, as shown in Fig. 8b. Next, brush electrode metal lithography was performed, and the metal was deposited (100 nm Pt) and patterned with a lift-off process, as shown in Fig. 8c. After metallization, two layers of 4.2-µm-thick polyimide were spun on and cured. Next, the droplet wall was patterned and etched with a timed etch, 3.6 µm into the polyimide, as shown in Fig. 8d. Following the wall etch, the overall outline of the microhydraulic layer, including the fluidic vias, was patterned in a 16 µm resist. The outline was etched just 1.5 µm short of the silicon–polyimide interface (Fig. 8e). This etch was critical, as the thinned regions of the polyimide must be less than 2 µm to be properly etched in the next section and more than 1 µm to not rip during the peel process. Next, the regions between the die were patterned, and die streets were etched all the way to the silicon interface. Finally, the polyimide was coated with a 20 nm film of fluoropolymer (Cytop Type A, Asahi Glass Co.), cured, and patterned with the hydrophobic resist pattern, as shown in Fig. 8f. Importantly, the hydrophilic pattern included all the regions in the outline pattern, so when the hydrophilic etch was performed in the next section, it fully separated the parts in the outline regions and formed the fluidic vias.

### Part 2: Die-based processing

Subsequent processing was performed on custom-built tools in the microfluidics laboratory on individual wafer dies. After cleaving the wafer, each polyimide die was peeled off, as shown in Fig. 8g. Peeling stopped 2 mm from the end of the die, leaving it partly attached to the silicon. A fluoropolymer solution (Cytop Type A, Asahi Glass Co., 0.6% in FC40, Sigma-Aldrich) was then injected into the peeling front, and the polyimide film was laid back down and then lifted again to wet the back with fluoropolymer. A video of this process is available in the Supplementary Section. After the layer was lifted again, excess fluoropolymer solution was drained, and the fluoropolymer film was annealed. The process was repeated with a second fluoropolymer (1% FluoroPel, Cytonix). Two fluoropolymer coats are required because FluroPel did not adhere to polyimide, and Cytop Type A did not electrowet properly. A sequential combination of the two resulted in a 14 nm film with acceptable adhesion and excellent electrowetting. Once both coats were applied and annealed, the polyimide layer was completely peeled and removed from the silicon die, and the silicon die was plasma etched to remove the fluoropolymer. Once the silicon die was free of fluoropolymer, the peeled edge of the polyimide film was placed back in the original position. Refined bee’s wax (Sigma-Aldrich) was melted into the interface between the wafer and the polyimide film, and the film was laid down for the third and final time. A video of this process is also available in the Supplementary Section. At the end of the peel process, the polyimide film had an electrowetting fluoropolymer added to the electrode side and a thin layer of release wax separating the silicon and the fluoropolymer. The die was then etched to remove the final 2 µm of polyimide from the layer outline and from the fluidic vias of the layers, as well as to remove the fluoropolymer from the exposed hydrophilic droplet regions. This etch had to be low temperature as not to melt the wax. Resist was then stripped in acetone, and the layers were wetted with a solution of water containing 8 M LiCl. Each rail was then pressurized with a micropipette (TIP10TW1-L, World Precision Instruments) attached to a monometer to a pressure of 12 cm of 8 M LiCl. Pressurization gave the droplets a well-defined curvature, as shown in Fig. 8h. After wetting and pressurization, individual microhydraulic layers were lifted off the wax by attaching a small piece of tape to the tab (Kapton tape with silicone adhesive). The tape adhesive and any residual wax were removed by cleaning in decane and dodecane. A fully released microhydraulic layer is shown in Fig. 8i.

Before assembly, the base layer was removed from the silicon wafer and transferred onto a clean glass slide to minimize parasitic capacitances. A drop of oil was placed on the base, and the first microhydraulic layer was aligned and pushed into position. This operation was performed with the layer at a small angle, ~10° to the base horizontal plane. As the layer was pushed off the aligner, it gently folded onto the base. Once it was sufficiently close, it self-aligned due to surface tension forces. Each subsequent layer was assembled the same way, as shown in the final step of a five-layer assembly in Fig. 8j. Before testing, each rail was pressurized to a Laplace pressure of 24 cm of 8 M LiCl using a micropipette through the top fluidic vias. The actuator was then placed in a dodecane bath for testing, as shown in Fig. 8k. Testing was performed using a National Instruments PXI system customized with a bank of high voltage amplifiers.

## Conclusion

Multilayer microhydraulic actuators demonstrate a unique combination of key concepts that enable precise, high-power, low-voltage capacitive actuation. Layering allows for a large actuation volume with low voltage at each layer. Stepping allows for high force and power, as well as a fine digital resolution of the motion. Different configurations of layers allow for actuators with multiple speed and force profiles without the use of gears. In a high-force configuration, multilayer microhydraulic actuators have a torque density that far exceeds that of inductive motors and is significantly greater than biological joints. A high-torque density results in a fast reaction time, with the actuators able to accelerate to full power and speed in <125 µs. These characteristics make multilayer microhydraulic actuators particularly well suited for robotics, where fast reactions to a dynamic environment are important. The technology may excel particularly well at the small scale where inductive motors are inefficient and find use in small robotic joints, microrobotics, robotic surgery^23^, and programmable materials^24^.

## Supplementary information

Process details

Five layer speed configuration actuation

Five layer force configuration actuation

Five layer linear speed configuration actuation

High speed actuation

Low speed actuation

Low-mag actuation

Peel and lay-lift process

Wax process

Wetting process
