# Supplementary material for: Multilayer microhydraulic actuators with speed and force configurations
Source: Microsyst Nanoeng. 2021 Mar 11;7:22. doi: 10.1038/s41378-021-00240-7 (PMC8433320; doi:10.1038/s41378-021-00240-7)
Supplement: Supplementary file 1 — Process details [file 41378_2021_240_MOESM1_ESM.docx]

**Supplemental Section – Process Details**

|  | **Wafer Based Processing** |  |
| --- | --- | --- |
| Step | Process | Parameter |
|  |  |  |
| 1 | Front Protect Coat | i-line resist 1.1 um thick |
| 2 | Scribe | Laser scribe on back |
| 3 | Clean | ACT CMI, 80C 5m 25C 5m |
| 4 | Clean | Sulfuric Acid, Hydrogen peroxide |
| 5 | 1.1 um Polyimide | PI2611 (2:1) 1165, 60s 3000rpm |
| 6 | Polyimide Bake | 120C 3m |
| 7 | Polyimide Cure | Ramp from 90C to 250C 2h |
| 8 | Ti/Al/Ox (M) evaporate | Ti 10nm, Al 60nm, SiO2 30nm |
| 9 | M Litho Coat | i-line resist 1.1 um thick |
| 10 | M Litho Expose | Expose 1300 J/m2 (M LEVEL MASK) |
| 11 | M Litho Develop | 60s Double Puddle |
| 12 | M Etch | EADBA-MHAMET06 , No DI |
| 13 |  | SiO2 etch Chamber A |
| 14 |  | Resist Strip Chamber D |
| 15 |  | Al etch Chamber B |
| 16 |  | SiO2 etch Chamber A |
| 17 | 0.9 um Polyimide | PI2611 (2:1) 1165, 80s 3000rpm |
| 18 | Polyimide Bake | 120C 3m |
| 19 | Polyimide Cure | 250C 2h |
| 20 | V Litho Coat | i-line resist 1.8 um thick |
| 21 | V Litho Expose | 5000 J/m2 (V LEVEL MASK) |
| 22 | V Litho Develop | 60s Double Puddle |
| 23 | Reflow Resist | 15 m 120C |
| 24 | V Etch | Polyimide etch (300s) |
| 25 | Resist Strip | ACT CMI, 80C 5m 25C 5m |
| 26 | S Under Resist Coat | i-line resist 1.1 um thick |
| 27 | S First Expose | 5000 J/m2 (BLANK MASK) |
| 28 | S Litho Coat | i-line resist 1.8 um thick |
| 29 | S Litho Expose | 5000 J/m2 F 0um (S LEVEL MASK) |
| 30 | S Litho Develop | 60s Double Puddle |
| 31 | Evaporate Ti/Pt | Ti 10nm Pt 100nm Pt |
| 32 | Liftoff clean | 1h/5s/5m/QDR/1h/5s/10m/QDR |
| 33 |  | 1h Acetone |
| 34 |  | 5s Ultrasonic in Acetone |
| 35 |  | 5m IPA |
| 36 |  | DI Rinse |
| 37 |  | 1h Acetone with occasional swabbing |
| 38 |  | 1h Acetone |
| 39 |  | 5s Ultrasonic in Acetone |
| 40 |  | 5m IPA |
| 41 |  | DI Rinse |
| 42 |  | Spin Rinse Dry |
| 43 | Electrical test | Verify metal connectivity and isolation |
| 44 | 4.3 um Polyimide | PI2611, 60s 3000rpm |
| 45 | Polyimide Bake | 120C 3m |
| 46 | Polyimide Cure | 250C 2h |
| 47 | 4.3 um Polyimide | PI2611, 60s 3000rpm |
| 48 | Polyimide Bake | 120C 3m |
| 49 | Polyimide Cure | 300C 2h |
| 50 | F Litho Coat | AZ1529, 30s 1400rpm |
| 51 | F Litho Expose | 10k J/m2 (F LAVEL MASK) |
| 52 | F Litho Develop | 120s Double Puddle |
| 53 | F Etch | Polyimide etch (400s) |
| 54 | F Strip | ACT CMI, 80C 5m 25C 5m |
| 55 | X Litho Coat | Spray Coat Resist 8um |
| 56 | X Litho Coat | Spray Coat Resist 8um |
| 57 | X Litho Expose | 15k J/m2, Focus +1.5um (X LEVEL MASK) |
| 58 | X Litho Develop | 480s 8 puddles |
| 59 | X Etch | Polyimide Etch (600s) |
| 60 | X Strip | ACT CMI, 80C 5m 25C 5m |
| 61 | C Litho Coat | Spray Coat Resist 8um |
| 62 | C Litho Expose | Use 10k J/m2, F=0um (C LEVEL MASK) |
| 63 | C Litho Develop | 480s 8 puddles |
| 64 | C Etch | Polyimide etch (350s) |
| 65 | C Strip | ACT CMI, 80C 5m 25C 5m |
| 66 | Stress Optimization Bake | 90C to 190C 2h |
| 67 | Coat Cytop | CYTOP Type A (0.6%) in FC3283, 1000 rpm 5s, 0rpm 10m |
| 68 | Bake Cytop | 170C 3m |
| 69 | H Litho Coat | Spray Coat Resist 8um |
| 70 | H Litho Expose | H Mask, 4k J/m2, F=0um |
| 71 | H Litho Expose | X Mask, 4k J/m2, F=-6um |
| 72 | H Litho Develop | 480s 8 puddles |
| 73 | H Litho Expose | H Mask, 2k J/m2, F=0um |
| 74 | H Litho Develop | 120s Double Puddle |

|  | **Assembly** |  |
| --- | --- | --- |
| Step | Process | Parameter |
|  |  |  |
| 75 | Dice wafers | 5x5 cm square |
| 76 | Peel polyimide | 0.1 mm/s 60deg inclination, with DI, 21C |
| 77 | Neutralization Anneal/Cooldown | 40C 20m/10m |
| 78 | CYTOP Lay/Lift | 0.05mm/s 0.6% CYTOP A in FC40, 30C |
| 79 | CYTOP anneal | 15m 55C |
| 80 | FluoroPel Lay/Lift | 0.05mm/s 1% FluoroPel, 30C |
| 81 | FluoroPel anneal | 15m 55C |
| 82 | Silicon wafer plasma clean | 10m 300W |
| 83 | Wax Apply | Refined beeswax, 3.2 mg, 45C |
| 84 | Wax, melt and laydown | 0.05 mm/s, 65C |
| 85 | Oxygen plasma etch | 50W 12m x4, Low Temperature Etch |
| 86 | Surface descum | O2 Plasma 300W 1m, 100W 2m |
| 87 | Resist strip / Clean | Acetone 3m, 5s UltraSonic, 1m Methanol |
| 88 | Water phase apply and pressurize | 8M LiCl at 12 cm |
| 89 | Wax Release | Peel with Tape on Tab |
| 90 | Clean Wax and Tape | Decane, Dodecane |
| 91 | Assembly | Layer assembly |
| 92 | Test | Actuator test |
